# Antimicrobial agent susceptibilities of *Legionella pneumophila* MLVA-8 genotypes

**DOI:** 10.1038/s41598-019-42425-1

**Published:** 2019-04-16

**Authors:** Yehonatan Sharaby, Orna Nitzan, Ingrid Brettar, Manfred G. Höfle, Avi Peretz, Malka Halpern

**Affiliations:** 1 Department of Evolutionary and Environmental Biology, Faculty of Natural Sciences, University of Haifa, Haifa, Israel; 2 Faculty of Medicine, Bar-Ilan University, Galilee, Safed, Israel; 3 Clinical Microbiology Laboratory, Baruch Padeh Poriya Medical Center, Tiberias, Israel; 4 Department of Vaccinology and Applied Microbiology, Helmholtz Centre for Infection Research (HZI), Braunschweig, Germany; 5 Department of Biology and Environment, Faculty of Natural Sciences, University of Haifa, Oranim, Tivon, Israel

## Abstract

*Legionella pneumophila* causes human lung infections resulting in severe pneumonia. High-resolution genotyping of *L. pneumophila* isolates can be achieved by multiple-locus variable-number tandem-repeat analysis (MLVA-8). *Legionella* infections in humans occur as a result of inhalation of bacteria-containing aerosols, thus, our aim was to study the antimicrobial susceptibilities of different MLVA-8 genotypes to ten commonly used antimicrobial agents in legionellosis therapy. Epidemiological cut-off values were determined for all antibiotics. Significant differences were found between the antimicrobial agents’ susceptibilities of the three studied environmental genotypes (Gt4, Gt6, and Gt15). Each genotype exhibited a significantly different susceptibility profile, with Gt4 strains (Sequence Type 1) significantly more resistant towards most studied antimicrobial agents. In contrast, Gt6 strains (also Sequence Type 1) were more susceptible to six of the ten studied antimicrobial agents compared to the other genotypes. Our findings show that environmental strains isolated from adjacent points of the same water system, exhibit distinct antimicrobial resistance profiles. These differences highlight the importance of susceptibility testing of *Legionella* strains. In Israel, the most extensively used macrolide for pneumonia is azithromycin. Our results point at the fact that clarithromycin (another macrolide) and trimethoprim with sulfamethoxazole (SXT) were the most effective antimicrobial agents towards *L. pneumophila* strains. Moreover, legionellosis can be caused by multiple *L. pneumophila* genotypes, thus, the treatment approach should be the use of combined antibiotic therapy. Further studies are needed to evaluate specific antimicrobial combinations for legionellosis therapy.

## Introduction

*Legionella pneumophila* has been found worldwide to be a relatively common pulmonary pathogen of severe community-acquired or nosocomial pneumonia^1–3^. *Legionella* infections in humans occur via inhalation of bacteria-containing aerosols, thus, the source of *Legionella* infection in humans is the environmental strains. Because of the ability of *Legionella* spp. to survive and multiply in human macrophages, they are susceptible to intracellularly active antimicrobial agents^4,5^. Currently, fluoroquinolones, macrolides, and rifampicin are the most commonly used antimicrobials in the treatment of legionellosis^4–7^. However, mortality rates of 10–15% are usually reported in legionellosis patients and death may occur despite antimicrobial agent therapy^6,8^. Furthermore, the presence of antimicrobial agents in the environment may promote the evolution of microbial resistance mechanisms^9^. This is particularly important for *Legionella* spp. that colonize most man-made water systems, where they may be exposed to antimicrobial agents of various artificial origins, or even to those secreted by other microorganisms^10^.

Although 25 of the 59 described *Legionella* species have been implicated in human disease^11–13^, the vast majority of cases are caused by *L. pneumophila* strains, most of which belong to serogroup 1^14–16^. Consequently, isolates of this common serogroup should be genotyped and further differentiated in order to evaluate the efficacy of antimicrobial agents in their treatment. Azithromycin is the most common macrolide used for treatment of community-acquired pneumonia in Israel. However, higher minimal inhibitory concentration (MIC) values have been reported for azithromycin compared with other macrolides for *L. pneumophila* serogroup 1. Thus, it is important to assess the susceptibility patterns of *L. pneumophila* in Israel as recommended in other countries^17^.

Multiple-locus variable-number tandem-repeat analysis (MLVA) was implemented by Pourcel *et al*.^18,19^ and approved by the European Centre for Disease Prevention and Control^19^. The method relies on the variability found in some tandemly repeated DNA sequences (VNTR) that represent sources of genetic polymorphism (Supplementary Fig. S1). This high-throughput typing method is used for epidemiological investigations of the origin of legionellosis cases since it allows rapid systematic typing of any new isolate and inclusion of data in shared databases^19–21^.

Recently, Rodríguez-Martínez *et al*.^22^ and Sharaby *et al*.^23,24^ showed that the level of genotypes (analyzed by MLVA-8) should be addressed in order to get insights into ecological traits of *L. pneumophila* strains inhabiting drinking water distribution systems (DWDSs). These studies showed that different sites of the same DWDS are dominated by different *L. pneumophila* MLVA genotypes. Analysis of the three dominant genotypes showed that they could be addressed as different ecotypes with a distinct temperature range, growth kinetics, virulence and abundances at their site of dominance^22–24^.

The aim of the current study was to analyze and compare the antimicrobial susceptibilities of different *L. pneumophila* genotypes to commonly used antimicrobial agents in legionellosis therapy. As far as we know, results from susceptibility testing of environmental and clinical *L. pneumophila* isolates have never been published in Israel. Since humans are infected with *Legionella* by inhaling *Legionella*-contaminated water aerosols, it is important to study the resistances of environmental strains to antibacterial agents and not only the clinical isolates. We determined the antimicrobial susceptibility profile for different *L. pneumophila* MLVA-8 genotypes. As each pneumonia patient can be infected by a mixture of *L. pneumophila* strains^25,26^, studying the antimicrobial susceptibility profiles of different environmental and clinical *L. pneumophila* strains is of great importance as it may shed light on the distribution of resistance to antimicrobial agents and assist in determining an accurate and efficient treatment for future legionellosis patients.

## Methods

### *L. pneumophila* strains

We studied the susceptibility of 93 environmental and 12 clinical strains to 10 antimicrobial compounds that are commonly used for legionellosis (Table 1). These strains were isolated from a drinking-water distribution system (DWDS) as part of a study conducted in northern Israel for two years (2013–2014, between coordinates 32°42′43.17″N, 35°6′28.66″E). During the sampling campaign, we sampled *Legionella* spp. seasonally from the drinking water systems of seven buildings. *Legionella* was isolated from water and biofilm samples according to ISO 11731:2004 and 11731:2017^27,28^ as described by Rodriguez-Martinez *et al*.^22^. In addition, we studied the susceptibility to antimicrobial agents of twelve clinical strains that were isolated from sputum samples of hospitalized pneumonia patients at Poriya and Rambam hospitals in northern Israel, between April 2013 and September 2014^23^.

**Table 1:** *Legionella pneumophila* genotypes used in the current study.

Sampling point	MLVA-8 Genotypes (n)	Sequence type (ST), Serogroup (Sg)	MLVA-8 genotype (Lpms)							
			(1)	(3)	(13)	(17)	(19)	(33)	(34)	(35)
**Environmental strains**										
A	Gt15 (11)	NA, Sg3	**9**	8	**8**	2	**5**	**2**	**2**	**21**
C, D	Gt4 (64)	ST1, Sg1	**7**	**7**	**10**	2	4	**4**	**2**	**17**
E, F, G	Gt6 (16)	ST1, Sg1	**7**	**7**	**10**	2	4	**4**	**2**	**18**
D	Gt18 (1)	ST1, Sg1	**7**	**7**	**7**	2	4	**4**	**2**	**17**
E	Gt3 (1)	NA, Sg1	**7**	**7**	**10**	2	4	**4**	**2**	**0**
**Clinical strains**										
Hospital	Gt4 (4)	ST1, Sg1	**7**	**7**	**10**	2	4	**4**	**2**	**17**
Hospital	Gt6 (2)	ST1, Sg1	**7**	**7**	**10**	2	4	**4**	**2**	**18**
Hospital	Gt19 (1)	ST1, Sg1	**7**	**7**	**10**	**1**	4	**4**	**2**	**17**
Hospital	Gt20 (1)	ST1, Sg1	**7**	**7**	**10**	2	4	**4**	**3**	**17**
Hospital	Gt22 (2)	ST59, Sg1	8	8	**10**	2	**5**	**4**	1	**13**
Hospital	Gt24 (2)	ST93, Sg1	8	8	11	2	**0**	1	1	3
**Reference strain**										
Philadelphia-1	Gt64	ST36, Sg1	**8**	**8**	**11**	**2**	**4**	**1**	**1**	**3**

Overview of the studied genotypes and their MLVA-8 allelic profiles; number of tandem repeats observed for each *L. pneumophila* minisatellite locus (Lpms). The indicated sampling points in the drinking-water network were representative for the whole network. The water flow direction was from sampling point A to G. For more details regarding the sampling points please see Rodríguez-Martínez *et al*.^22^.

Allelic repeats profiles for the reference strain were obtained from Pourcel *et al*.^19^. Highlighted in bold are differences in tandem repeats for each genotype compared to the type strain *L. pneumophila* Philadelphia-1.

### Reference strains

*L. pneumophila* subsp. *Pneumophila* sg. 1 of the American Type Culture Collection (ATCC 33152) was used as the reference strain. In addition, *Staphylococcus aureus* (ATCC 29213) and *Escherichia coli* (ATCC 25922) were also selected for validation of susceptibility testing results (Table 2). The selected strains were kept frozen at −80 °C prior to analysis.

**Table 2:** Minimal inhibitory concentrations (µg/ml) of each antimicrobial agent, towards the reference strains.

	*L. pneumophila* (ATCC 33152)	*E. coli* (ATCC 25922)	*S. aureus* (ATCC 29213)
Ciprofloxacin	0.032	0.25	N.D.
Moxifloxacin	0.032	0.25	N.D.
Levofloxacin	0.032	0.064	0.25
Tigecycline	0.064	0.25	N.D.
Doxycycline	0.032	N.D.	0.023
Azithromycin	0.032	N.D.	0.023
Erythromycin	0.047	N.D.	0.25
Clarithromycin	0.047	N.D.	0.032
Rifampicin	0.023	N.D.	0.25
SXT^a^	0.023	0.19	2.0

^*^SXT, Trimethoprim and sulfamethoxazole; N.D., not determined.

### *L. pneumophila* molecular typing

**Figure 1:**
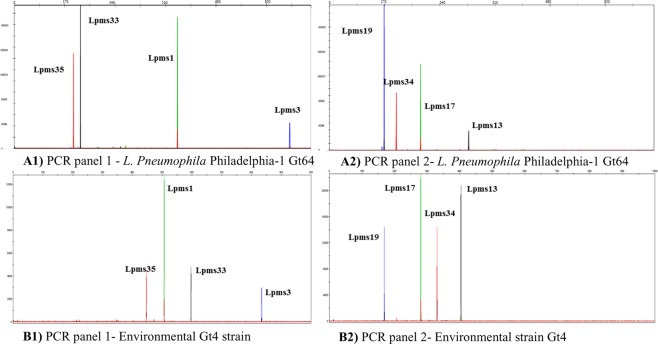
Representative electropherograms of MLVA-8 PCR products of multiplex PCR, separated by capillary electrophoresis and identified according to their sizes and colors. Electropherograms correspond to MLVA-8 PCR products of panel 1 and panel 2 of (A) Genotype 64 (Gt64) *L. pneumophila* Philadelphia-1 and (B) Genotype 4 (Gt4) an environmental strain. Repeats number at each Lpms locus was identified by color and peak size by GeneMapper software, version 3.7 (Applied Biosystems). Repeat profiles were then compared with the MLVA-8 database for *Legionella* (http://microbesgenotyping.i2bc.paris-saclay.fr/databases/view/887). The workflow chart of the MLVA-8 analysis is explained in Supplementary Fig. S1 and in the Methods section 2.3.

Genotyping of the strains was achieved by Multi Locus Variable number of tandem repeat Analysis using eight loci (MLVA-8) as described by Pourcel *et al*.^21,22^, Kahlisch *et al*.^29^ and Pecellin^30^ (Fig. 1). Briefly, 1 × 10^−2^ ng of DNA template was used in 25 µl PCR reactions containing 1 Multiplex PCR Master Mix (Qiagen, Hilden, Germany) and 1.25 pmol of each primer (VIC®-, NED®-, FAM-, and NET-labeled forward primers from Applied Biosystems, Foster City, CA). Primers sequences, position, and repeat sizes at each variable number of tandem repeats (VNTR) locus are listed in Supplementary Table S1. After amplification, PCR products were pooled and denatured. Amplicons were then separated by size using fluorescent capillary electrophoresis, a powerful separation technique based on the differential size-dependent migration of DNA molecules in an electric field. Fluorescent capillary electrophoresis of the multiplex PCR products was performed with a 3730 × L sequencer (Applied Biosystems) as described in Nederbragt *et al*.^21^. We used a pre-run voltage of 8.0 kV, run voltage of 8 kV, injection voltage of 1.8 kV and injection time of 15 sec. Each *L. pneumophila* mini-microsatellite locus (Lpms) was identified by color and assigned a size by GeneMapper software, version 3.7 (Applied Biosystems), using settings for VNTR analysis as shown in Fig. 1. The final repeat profile was then compared with the MLVA-8 database for *Legionella* (http://microbesgenotyping.i2bc.paris-saclay.fr/databases/view/887). The genotypes of 63 environmental strains were reported in detail by Rodríguez-Martínez *et al*.^22^. Additionally, in this study we genotyped 30 more environmental strains isolated from the same sampling campaign. The genotypes for the 12 clinical isolates were reported in Sharaby *et al*.^23^. The details of the studied strains regarding their isolation source, their serogroup (sg), sequence type (ST), and their genotypes (Gt) are listed in Table 1.

### Determination of MICs

Isolates from buffered charcoal yeast extract agar supplemented with α-ketoglutarate (BCYE-α) plates (BD Diagnostics Sparks, MD) were suspended in 0.85% NaCl solution to a 0.5 McFarland standard and subjected to MIC test strip (Liofilchem s.r.l., Italy) on BCYE-α plates. A sterile cotton swab was soaked in the inoculum suspension of each isolate. Each swab was then streaked over the entire BCYE-α agar plate surface. Plates were left to dry for 10 minutes so that the surface was completely dry before applying the Epsilometer test gradient strip. The following antimicrobial agents were used: Azithromycin, Clarithromycin, Ciprofloxacin, Moxifloxacin, Rifampicin, Tigecycline, Doxycycline, Levofloxacin, Erythromycin, and Trimethoprim-sulfamethoxazole. The MICs were read after 48 hours of incubation at 35 ± 1 °C at 2.5% CO_2_. The MIC of each antimicrobial agent was taken as the lowest concentration of the antimicrobial agent at which the zone of inhibition intersected the strip. MIC tests for *L. pneumophila* isolates were repeated in triplicates. Epidemiological Cut-off values (ECOFFs) were determined according to the European Committee on Antimicrobial Susceptibility Testing (EUCAST) guidelines for *Legionella pneumophila*^31^. Briefly, MIC values were fitted to the cumulative log-normal distribution using non-linear least squares regression in order to determine the ECOFF for each antimicrobial agent^31^.

### Statistical analysis

All statistical analyses were performed using IBM SPSS 22® and Primer7 software (Primer-e, Auckland, New Zealand). All tests were applied at a 95% level of confidence. Repeated-measures analysis of variance (ANOVA) was applied to study the differences between the MICs of different antimicrobials. Data sphericity was not violated (Mauchly’s test: *p* > 0.05). T-tests were applied in order to compare the antimicrobials’ MICs for strains isolated from environmental versus clinical sources, water versus biofilm and hot versus cold water. In addition, analysis of similarities (ANOSIM) was performed^32^ in order to compare the antimicrobial agent resistance profiles of environmental genotypes and clinical isolates taking into account all studied antimicrobial agents’ MIC values. The resemblance matrix was calculated using the Bray-Curtis index of association (Primer7 software). One-way ANOVA was used to determine whether significant differences exist in antimicrobial agents’ MICs between different MLVA-8 genotypes (Gt4, Gt6, and Gt15). All groups were normally distributed according to Shapiro-Wilk test (*p* > 0.05) and variances were equal between groups (Levene’s test: *p* > 0.05).

## Results

The susceptibilities of 93 environmental and 12 clinical *L. pneumophila* strains to 10 antimicrobial agents commonly used in legionellosis therapy were analyzed. The environmental strains that were studied here represent a subset of the strains belonging to three MLVA-8 genotypes (Gt) 4, 6, and 15^22^ that dominated a water network in northern Israel (Table 1). The clinical strains belonged to Gt4 and Gt6, Gt19, Gt20, Gt22, and Gt24 (Table 1). All strains except Gt15 were classified as serogroup 1. Gt15 strains were classified as serogroup 3 (Table 1). We used *L. pneumophila* subsp. *Pneumophila* sg 1 (ATCC 33152) as the reference strain. In addition, *Staphylococcus aureus* (ATCC 29213) and *Escherichia coli* (ATCC 25922) were also selected for validation of susceptibility testing results (Table 2). The MICs obtained for both *S. aureus* and *E. coli* were generally lower but within an order of magnitude compared to the findings of previous studies^33,34^.

**Figure 2:**
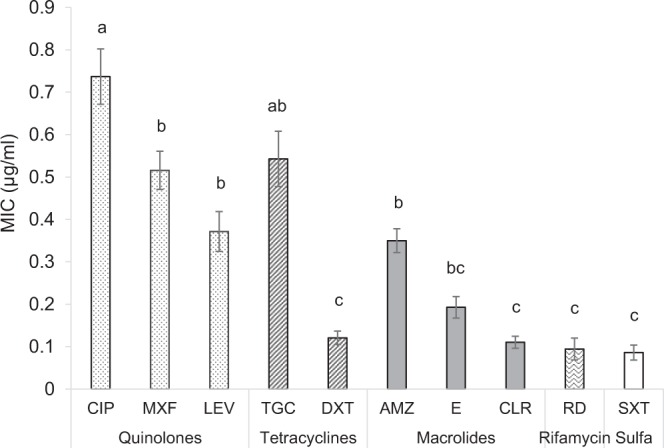
Minimal inhibitory concentrations (average ± standard error) of each studied antimicrobial agent towards *L. pneumophila* strains isolated from both clinical and environmental sources (n = 105). Ciprofloxacin – CIP, moxifloxacin – MXF, levofloxacin – LEV, tigecycline – TGC, doxycycline – DXT, azithromycin – AMZ, erythromycin – E, clarithromycin – CLR, rifampicin – RD, trimethoprim & sulfamethoxazole – SXT. Bars connected by different letters are significantly different by repeated-measures ANOVA with Tukey’s HSD post-hoc test with a confidence interval of 95%.

Overall, significant differences were observed in *L. pneumophila* sensitivities to different antimicrobial agents (Repeated measures ANOVA: F_9,792_ = 15.27, *p* < 0.001). Minimal inhibitory concentrations (MICs) of antimicrobial agents from the fluoroquinolone family were significantly higher compared to those of the macrolides, doxycycline, rifampicin, and trimethoprim & sulfamethoxazole (SXT) (Fig. 2, Table 3). The lowest MICs were observed after exposure to SXT, yet no significant differences were found between the MICs of SXT, erythromycin, Clarithromycin, and Rifampicin (Fig. 2). The highest MIC was found for ciprofloxacin (0.74 ± 0.06 µg/ml) and it was significantly higher than the MICs of moxifloxacin and levofloxacin, which are fluoroquinolones (0.52 ± 0.04 and 0.37 ± 0.04 µg/ml, respectively). MIC_50_ values yielded similar results with the highest MIC_50_ found for ciprofloxacin (0.75 µg/ml) and the lowest for SXT with 0.023 µg/ml (Tables 3, 4).

**Table 3:** The accumulated percentages (%) of all the tested strains (93 environmental and 12 clinical *L. pneumophila* isolates), that were inhibited at each concentration of the different antimicrobial agents (µg/ml).

µg/ml	0.016	0.023	0.032	0.064	0.094	0.125	0.19	0.25	0.38	0.5	0.75	1	1.5	2
Ciprofloxacin	2	2	18	18	21	21	28	39	40	47	53	81	92	100
Moxifloxacin			27	27	28	30	30	42	42	62	68	99	100	
Levofloxacin		9	43	50	51	51	51	53	56	73	88	96	100	
Tigecycline		2	40	43	43	43	43	43	43	66	74	88	94	100
Doxycycline		10	69	73	73	73	82	87	87	100				
Azithromycin			13	19	26	31	36	49	59	87	96	100		
Erythromycin		5	42	49	50	54	72	79	81	96	98	100		
Clarithromycin	1	5	49	72	74	75	81	93	93	100				
Rifampicin	48	51	77	87	89	89	89	89	89	95	98	100		
SXT*	42	53	72	80	81	83	86	90	92	99	100			

^*^SXT, Trimethoprim and sulfamethoxazole.

The values for each antimicrobial agent are the percentage of the strains that were inhibited in the above mentioned antimicrobial agent concentration, thus, MIC_50_ and MIC_90_ values can be read directly from this table. For example, SXT has a MIC_50_ of between 0.016–0.023 µg/ml and a MIC_90_ of 0.25 µg/ml.

**Table 4:** MIC_50_, MIC_90_, MIC range and ECOFF values (µg/ml) of the 10 tested antimicrobial agents for all *L. pneumophila* strains (n = 105).

Drug	MIC_50_	MIC_90_	Range	ECOFF*
Ciprofloxacin	0.75	1.5	0.019–2.0	4.0
Moxifloxacin	0.5	1.0	0.032–1.5	4.0
Levofloxacin	0.075	1.0	0.023–1.5	1.0
Tigecycline	0.5	1.5	0.023–2.0	0.5
Doxycyline	0.032	0.5	0.023–0.5	0.5
Azithromycin	0.38	0.75	0.032–1.0	2.0
Erythromycin	0.094	0.5	0.023–1.0	0.5
clarithromycin	0.064	0.25	0.025–0.5	0.5
Rifampicin	0.023	0.5	0.003–1.0	0.063
SXT**	0.023	0.25	0.003–0.75	0.5

MIC_50_, MIC_90;_ Lowest concentration of the antimicrobial agents at which 50% and 90% of the isolates were inhibited, respectively. ^*^ECOFF, epidemiological cut-off values. ^**^SXT, Trimethoprim and sulfamethoxazole.

No significant differences were detected between the susceptibilities of environmental strains isolated from bulk water (n = 58) *vs*. biofilms (n = 35) (t-tests: df = 91, p > 0.1, for all studied antimicrobial agents). Moreover, t-tests did not detect any significant differences in antimicrobial agent resistances of strains isolated from cold (n = 32) *vs*. hot water (n = 26) (t-tests: df = 56, p > 0.1, for all studied antimicrobial agents). For additional details, see Supplementary Table S2. In contrast, t-tests revealed significant differences in antimicrobial agent susceptibilities of environmental *vs*. clinical (e.g., isolated from patients’ sputum) strains. Environmental strains were significantly more resistant towards five of the 10 studied antimicrobial agents compared to *L. pneumophila* strains from clinical sources (Table 5). The largest difference was found after exposure to ciprofloxacin; MIC_50_ of ciprofloxacin was 1.0 µg/ml for the environmental strains and only 0.22 µg/ml for the clinical strains (Table 5). In addition, the MICs of tigecycline, clarithromycin, rifampicin, and SXT were also significantly higher for the environmental strains compared to the clinical *L. pneumophila* strains (Table 5). In contrast, doxycycline was the only studied antimicrobial agent for which the clinical strains were more resistant, with MIC_50_ and MIC_90_ of 0.19 and 0.5 µg/ml compared to 0.25 and 0.032 µg/ml for the environmental strains, respectively (Table 5). In addition, analysis of similarities (ANOSIM) revealed significant differences between the antimicrobial agent resistance profiles of clinical and environmental *L. pneumophila* isolates (R = 0.62, *p* < 0.001). However, a comparison of Gt4 strains from clinical *vs*. environmental sources showed no significant differences in antimicrobial agent resistances (t-tests: df = 62, *p* > 0.05). This may be due to low sample size of the clinical Gt4 strains (n = 4).

**Table 5:** Antimicrobial MICs (µg/ml) for the clinical and the environmental *L. pneumophila* strains and for each of the environmental genotypes.

	Gt4 (n = 64)	Gt6 (n = 16)	Gt15 (n = 11)	^*^Environmental (n = 93)	Clinical (n = 12)
Ciprofloxacin	**2.0** (1)	**0.875** (0.22)	**1.0** (0.5)	**1.5** (1)	**0.475** (0.22)
Moxifloxacin	**1.0** (0.75)	**0.25** (0.0395)	**1.0** (0.5)	**1.0** (0.5)	**1.0** (0.5)
Levofloxacin	**1.0** (0.5)	**0.157** (0.032)	**0.75** (0.032)	**1.0** (0.064)	**0.75** (0.288)
Tigecycline	**1.5** (0.75)	**0.056** (0.032)	**0.5** (0.047)	**1.5** (0.5)	**0.5** (0.047)
Doxycycline	**0.25** (0.032)	**0.19** (0.032)	**0.5** (0.064)	**0.25** (0.032)	**0.5** (0.19)
Azithromycin	**0.75** (0.44)	**0.19** (0.0555)	**0.5** (0.25)	**0.75** (0.38)	**0.725** (0.375)
Erythromycin	**0.5** (0.079)	**0.25** (0.142)	**0.5** (0.19)	**0.5** (0.125)	**0.19** (0.032)
Clarithromycin	**0.25** (0.056)	**0.5** (0.064)	**0.5** (0.25)	**0.25** (0.064)	**0.064** (0.047)
Rifampicin	**0.5** (0.032)	**0.012** (0.006)	**1.0** (0.032)	**0.5** (0.032)	**0.006** (0.004)
SXT^**^	**0.25** (0.023)	**0.253** (0.032)	**0.5** (0.032)	**0.354** (0.032)	**0.023** (0.006)

^*^Environmental strains included two strains designated Gt3 and Gt18 in addition to the listed Gt4, Gt6, Gt15 strains. ^**^SXT, Trimethoprim and sulfamethoxazole.

MIC_90_ values are in bold and MIC_50_ values are presented in brackets.

### Environmental genotypes

**Figure 3:**
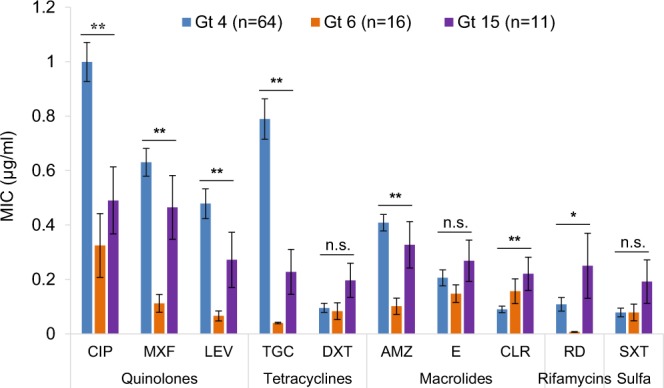
Minimal inhibitory concentrations (average ± standard error) of each studied antimicrobial agent for different MLVA-8 genotypes. Ciprofloxacin – CIP, moxifloxacin – MXF, levofloxacin – LEV, tigecycline – TGC, doxycycline – DXT, azithromycin – AMZ, erythromycin – E, clarithromycin – CLR, rifampicin – RD, trimethoprim & sulfamethoxazole – SXT. Asterisks represent significant differences by one-way ANOVA with Tukey’s post-hoc tests between genotypes at the 0.05* and 0.001** levels of confidence. n.s., not significant.

One-way ANOVA revealed significant differences in the resistance of the co-localized environmental genotypes (F_2,88_ = 128.73, *p* < 0.001). Gt4 strains were found to be significantly more resistant towards ciprofloxacin, moxifloxacin, levofloxacin, tigecycline, and azithromycin compared to strains belonging to Gt6 and Gt15 (Fig. 3 and Table 5). The highest MIC_90_ values were obtained for Gt4 strains after exposure to ciprofloxacin and tigecycline (2 µg/ml and 1.5 µg/ml, respectively). The MIC_90_ values of Gt6 strains were significantly lower compared to other genotypes after exposure to six out of the ten studied antimicrobial agents. The lowest MIC_90_ values for Gt6 strains were obtained with tigecycline and rifampicin (0.056 and 0.012 µg/ml, respectively); an order of magnitude lower compared to the MICs of Gt4 and Gt15 strains (Fig. 3 and Table 5). Gt15 strains were significantly more resistant to clarithromycin, rifampicin, and SXT (with MIC_90_ of 0.5, 1, and 0.5 µg/ml, respectively). In addition, analysis of similarities showed that different genotypes possess significantly different resistance profiles (ANOSIM: R = 0.287, *p* = 0.001).

## Discussion

MLVA is a useful genotyping method as it allows a good resolution within the highly health-relevant and abundant Sequence Type 1 (ST1) strains (Table 1). For example, genotypes 4 and 6 are both classified as ST1, and cannot be differentiated by the sequence-based typing method. Moreover, genotype 4 comprises the reference strain *L. pneumophila* Paris, which belongs to ST1^19^. Mercante and Winchell^12^ and McDade^35^ have suggested that the level of genotypes should be addressed in order to assess the health risks posed by the presence of different *L. pneumophila* strains in DWDSs. As far as we know this is the first study that compares susceptibilities of environmental *L. pneumophila* MLVA-8 genotypes to antimicrobial agents.

Recently, we have demonstrated that *L. pneumophila* dominated different sites of a small Israeli drinking water network, with MLVA-8 genotype related abundance regime^22^. These genotypes demonstrated different temperature-dependent growth kinetics and different cytotoxicity towards amoebae, macrophages and red blood cells^23,24^. Hence, here we show that these same isolates differed also in their susceptibilities to antimicrobial agents (Tables 1 and 5). MLVA-8 genotypes 4 and 6 strains exhibited distinct growth characteristics despite the fact that both are classified as ST1 by sequence-based typing. Gt4 strains were able to proliferate more rapidly in temperatures of 25–37 °C compared to genotypes Gt6 and Gt15 strains^23^. In addition, Gt4 strains were significantly more cytotoxic towards amoebae and macrophages under *in vitro* experimental conditions^24^. In the current study, Gt4 strains were significantly more resistant towards five out of the 10 antimicrobial agents that were studied, compared to Gt6 strains (Fig. 3). These findings suggest that ST1 strains belonging to Gt4 genotypes may pose a much more severe health risk compared to ST1 strains belonging to Gt6 (Tables 1 and 5). Our current findings indicate that these environmental genotypes, although colonizing the same niche in the drinking water system, should be addressed as different ecotypes since a high variability exists even among ST1 strains in terms of their antimicrobial resistance profiles.

Coscollá *et al*.^25^ observed mixed infections of *L. pneumophila* strains in outbreak patients. They analyzed sequence based typing profiles of uncultured respiratory samples and found evidence of a mixture of *Legionella* ST profiles in patients. They concluded that patients might be infected from the environment by more than one *L. pneumophila* strain. Recently, Mizrahi *et al*.^26^ also reported that a mix of *L. pneumophila* strains were identified from sputum samples of pneumonia patients. These findings, along with the results described here regarding the high variability of *L. pneumophila* genotypes’ antimicrobial agent resistances, emphasize the importance of high-resolution identification of different genotypes and their antimicrobial agent susceptibility profiles, especially in pneumonia patients. In such cases of mixed lung infections caused by multiple *L. pneumophila* genotypes, the application of combination of antibiotic therapy should be considered since it might provide better treatment outcomes. Dual combination antibiotic therapy was shown to improve treatment outcomes and survival in patients with severe community-acquired pneumonia caused by *Legionella* and other pathogenic bacteria^8,36^. Adding a macrolide or fluoroquinolone to a β-lactam was already recommended by the Infectious Diseases Society of America/American Thoracic Society guidelines^37^. For example, the combination of rifampicin with clarithromycin showed decreased mortality rates in patients^8^. In our study, both rifampicin and clarithromycin, were found to be very effective towards the three compared genotypes (Fig. 3 and Table 5). Therefore, their combination in treating mixed infections caused by several *L. pneumophila* genotypes may improve treatment outcomes compared to monotherapy. Further research with emphasis on different MLVA genotyping will allow more accurate assessments of the different antimicrobials’ efficacies in treatment of human infections.

It has been previously reported that performing E-test on BCYE-α agar may yield elevated MICs^38^. Nonetheless, it still provides a simple yet accurate method for routine and comparative susceptibility testing of *Legionella* spp. However, the MIC value itself, should not be directly translated to serum concentrations of these antimicrobial agents. Thus, it can be used for detecting antimicrobial resistances. Sufficient data to establish ECOFFs are currently not available^31^. In the current study, ECOFFs were determined according to the EUCAST guidelines for *L. pneumophila* susceptibility testing (Table 4)^31^. Our findings can be used in the future in the process of setting epidemiological cut off values^33^.

Antimicrobial agent susceptibility of *Legionella* strains isolated from drinking water sources was studied previously. Xiong *et al*.^39^ found that levofloxacin was the most effective drug against different *L. pneumophila* serogroups. Minocycline and doxycycline were also found to be effective. Torre *et al*.^34^ and Sikora *et al*.^40^ found that ciprofloxacin and rifampicin have good activity against environmental *L. pneumophila* sg 1 and sg 2–14. For the overall set of strains tested in the current study, we found that the most effective drugs towards *L. pneumophila* strains were doxycycline, clarithromycin, rifampicin, and SXT (Fig. 2). Moreover, the strains in the current study were found to be relatively resistant towards levofloxacin and ciprofloxacin (the most effective drugs according to Xiong *et al*.^39^ and Sikora *et al*.^40^, respectively).

Azithromycin (macrolides) and respiratory fluoroquinolones are the most commonly used antimicrobial agent treatments for community-acquired pneumonia^37,41–44^. Numerous public health agencies such as the Infectious Diseases Society of America (IDSA), the British Thoracic Society (BTS) and the Dutch Association of Chest Physicians recommend using fluoroquinolones (ciprofloxacin in particular), or azithromycin, as a preferred antimicrobial therapy for legionellosis cases^6,37,45^. Thus, it is of major importance to verify high susceptibility rates of *L. pneumophila* to these antimicrobial agents. We found significantly higher MIC values to fluoroquinolones compared with macrolides, which might justify empiric and definitive treatment with macrolides as first line treatment of *L. pneumophila* pneumonia in Israel (Fig. 2, Table 3). In contrast, other studies reported that quinolones have greater activity toward *L. pneumophila* compared with macrolides, with a reduced length of stay, and reduced time to clinical resolution^43,46^. Nevertheless, these differences probably resulted from differences in the susceptibility testing methods used^43,44,46,47^. In Israel, azithromycin is the most extensively used macrolide for treatment of community-acquired pneumonia as well as *L. pneumophila* pneumonia. In the current study, we found higher MIC values for azithromycin versus clarithromycin (Fig. 2 and Table 4). This is similar to the findings of studies conducted in southern Italy on the susceptibilities of *L. pneumophila* strains^17,34^.

Recently, Massip *et al*.^48^ showed that *lpeAB* genes encode components of a tripartite efflux pump implicated in resistance to azithromycin among other macrolides in *L. pneumophila*. In addition, Vandewalle-Capo *et al*.^49^ demonstrated that the reduced azithromycin susceptibility of ST1 strains was linked to the presence of these *lpeAB* genes. In our study, we found significant differences in resistance to azithromycin between co-localized genotypes, especially Gt4 and Gt6 strains, both belonging to ST1 (Fig. 3 and Table 5). This finding might justify further studies of antimicrobial agents’ clinical efficacy towards different genotypes and possibly a switch to treatment with clarithromycin. Moreover, in our study, the lowest MIC values were observed after exposure to trimethoprim – SXT (Fig. 2 and Tables 3, 4). These antimicrobial agents are not regularly used to treat *L. pneumophila* infections and thus, further research is needed to evaluate the efficiency of SXT for treating legionellosis.

*Legionella* patients are infected with the bacteria by inhaling water droplets containing *Legionella*. Thus, the source of the clinical strains is the environmental strains and it is important and useful to predict the onset of antimicrobial agent resistance in the environment before it is evidenced in clinical specimens^33,50^. In the current study, environmental strains were significantly more resistant towards five (ciprofloxacin, tigecycline, clarithromycin, rifampicin, and SXT) out of the 10 studied antibacterial agents, compared to strains of clinical source. Clinical strains were significantly more resistant only to Doxycycline compared to the environmental strains (Table 5). In addition, the antimicrobial resistance profiles of clinical and environmental strains differed significantly (Table 5). Earlier studies suggested that the presence of antimicrobial agents in the environment, and especially in man-made drinking-water distribution systems (DWDSs), might promote the evolution of microbial resistance mechanisms^9,10^.

Since only one case of person-to-person transmission of *Legionella* has been reported so far^51^, the human body is considered to be a “dead-end” for the evolution of this pathogen. Therefore, clinical strains probably do not transfer antimicrobial resistances to the environment and the environmental strains are the source for the clinical cases of *L. pneumophila* pneumonia infections. It is of great importance to adjust the antibacterial therapy for legionellosis patients to fit the susceptibilities of environmental strains that are present in DWDSs. Our results show that a considerable amount of variability exists in terms of antimicrobial resistances of environmental strains (Fig. 3 and Table 5). A rapid and reliable method for distinguishing between strains is necessary in order to determine the specific susceptibilities of environmental *L. pneumophila* genotypes.

Routine monitoring and susceptibility testing of environmental strains from DWDSs can allow detection of antimicrobial resistances acquisition. However, as reported in previous studies, there are difficulties in determining MICs for *Legionella* (for example, inactivation of some antibiotics by charcoal)^34,40^. Consequently, it is difficult to compare results obtained from different methods and establish ECOFF values. Therefore, highly efficient techniques are needed in order to isolate environmental *Legionella* strains from the environment and then test and monitor the acquisition of resistance in the environmental context of the network.

### In conclusion

We determined the antimicrobial agent susceptibility profiles for different *L. pneumophila* MLVA-8 genotypes. Gt4 strains belonging to ST1 were significantly more resistant towards Ciprofloxacin, Moxifloxacin, Levofloxacin, Tigecycline, and Azithromycin compared to strains belonging to Gt6 (also belonging to ST1), and Gt15 genotypes (Fig. 3). Our results demonstrate that although these environmental strains were isolated from adjacent points of the same drinking water system, they are distinct in terms of their antimicrobial agent susceptibilities as was also observed for their other physiological traits^23,24^. Evidence pointed out that pneumonia patients may acquire a mixture of *L. pneumophila* strains^25,26^. These, along with the results regarding the high variability of *L. pneumophila* genotypes’ antimicrobial resistance profiles, emphasize the importance of studying antimicrobial resistances of different *L. pneumophila* genotypes. Moreover, since the human body is considered a “dead-end” for the evolution of *Legionella*, it is important to study the antimicrobial resistances not only for clinical isolates, but also for the environmental strains that are the source of the clinical infections.

## Supplementary information

Supplementary Dataset 1
