# Supplementary material for: Antimicrobial agent susceptibilities of Legionella pneumophila MLVA-8 genotypes
Source: Sci Rep. 2019 Apr 16;9:6138. doi: 10.1038/s41598-019-42425-1 (PMC6468011; doi:10.1038/s41598-019-42425-1)
Supplement: Supplementary file 1 — Supplementary Dataset 1 [file 41598_2019_42425_MOESM1_ESM.docx]

# Antimicrobial agent susceptibilities of *Legionella pneumophila* MLVA-8 genotypes

Yehonatan Sharaby^1^, Orna Nitzan^2,3^, Ingrid Brettar^4^, Manfred, G. Höfle^4^, Avi Peretz^2,3#*^, Malka Halpern^1,5#*^

^1^Department of Evolutionary and Environmental Biology, Faculty of Natural Sciences, University of Haifa, Haifa, Israel.

^2^ Faculty of Medicine, Bar-Ilan University, Galilee, Israel.

^3^ Clinical Microbiology Laboratory, Baruch Padeh Poriya Medical Center, Israel.

^4^Department of Vaccinology and Applied Microbiology, Helmholtz Centre for Infection

Research (HZI), Braunschweig, Germany.

^5^Department of Biology and Environment, Faculty of Natural Sciences, University of Haifa, Oranim, Tivon, Israel.

^#^Equal contribution

**Supplementary Fig. S1.** Workflow chart of the MLVA-8 analysis. PCRs with fluorescently labeled primers for capillary electrophoresis (CE) enable the determination of the repeat numbers in VNTR loci through the analysis of the peaks in the electropherograms as shown in Figure 1. The different number of repeats at each VNTR locus is reflected in the MLVA profiles, which are specific for each strain. The MLVA database for *Legionella pneumophila* allows the comparison of allelic repeat profiles (<http://microbesgenotyping.i2bc.paris-saclay.fr/databases/view/887>) between strains.

**Supplementary Table S1.**

MLVA primers position and repeat size at each VNTR locus.

| **Primer^*^** | **Sequence (5′-3′)** | **Position^**^** | **Repeat size (bp)** | **Locus size (bp)^***^** |
| --- | --- | --- | --- | --- |
| Lpms1F | ACGAGCATATGACAAAGCCTTG | 3231397 | 45 | 565 |
| Lpms1R | CGGATCATCAGGTATTAATCGC | 3230833 |  |  |
| Lpms3F | CAACCAATGAAGCAAAAGCA | 3144593 | 96 | 941 |
| Lpms3R | AGGGGTTGATGGTCTCAATG | 3145533 |  |  |
| Lpms13L | CAATAGCATCGGACTGAGCA | 1645030 | 24 | 428 |
| Lpms13R | TGCCTGTGTATCTGGAAAAGC | 1644603 |  |  |
| Lpms17F | CAGCTCACCCCGTATCACTT | 930888 | 39 | 278 |
| Lpms17R | TAACATCAATGACCGCGAAA | 931165 |  |  |
| Lpms19F | GAACTATCAGAAGGAGGCGAT | 913318 | 21 | 173 |
| Lpms19R | GGAGTTTGACTCGGCTCAGG | 913490 |  |  |
| Lpms33F | ACCACAGCAGTTTGAACATAAT | 2578624 | 125 | 227 |
| Lpms33R | GGGAGAAGTTATAGATCTATTCG | 2578850 |  |  |
| Lpms34F | GAAAAGGAATAAGGCGCAGCAC | 2661871 | 125 | 209 |
| Lpms34R | AAACCTCGTTGGCCCCTCGCTT | 2662079 |  |  |
| Lpms35F | CTGAAACAGTTGAGGATGTGA | 1428273 | 18 | 202 |
| Lpms35R | TTATCAACCTCATCATCCCTG | 1428474 |  |  |

^*^Lpms- *Legionella pneumophila* minimicrosatellite
^**^Primers Positions numbering (5′ end) according to the reference sequence Philadelphia-1^1^.
^***^For the reference strain *Legionella pneumophila* Philadelphia-1 (ATCC33152).

**Supplementary Table S2.**

Antimicrobial MICs (µg/ml) for environmental *L. pneumophila* strains isolated from cold and hot water and from biofilm. MIC_90_ values are in bold and MIC_50_ values are presented in brackets. See also Table 5.

|  | **Cold water (n=32)** | **Hot water (n=26)** | **Biofilm (n=35)** |
| --- | --- | --- | --- |
| **Ciprofloxacin** | **1.5** (1) | **1.5** (0.625) | **1.5** (1) |
| **Moxifloxacin** | **1** (0.75) | **1** (0.41) | **1** (0.5) |
| **Levofloxacin** | **1** (0.064) | **0.875** (0.047) | **1** (0.5) |
| **Tigecycline** | **1.95** (0.5) | **1** (0.5) | **1.5** (0.5) |
| **Doxycyline** | **0.475** (0.032) | **0.25** (0.04) | **0.226** (0.032) |
| **Azithromycin** | **0.75** (0.38) | **0.625** (0.275) | **0.65** (0.38) |
| **Erythromycin** | **0.488** (0.079) | **0.5** (0.158) | **0.5** (0.064) |
| **clarithromycin** | **0.5** (0.064) | **0.25** (0.047) | **0.25** (0.047) |
| **Rifampicin** | **0.5** (0.032) | **0.5** (0.032) | **0.082** (0.012) |
| **SXT^*^** | **0.367** (0.032) | **0.22** (0.023) | **0.4** (0.032) |

^*^SXT – Trimethoprim and sulfamethoxazole.
